# The E3 ligase RNF5 restricts SARS-CoV-2 replication by targeting its envelope protein for degradation

**DOI:** 10.1038/s41392-023-01335-5

**Published:** 2023-02-03

**Authors:** Zhaolong Li, Pengfei Hao, Zhilei Zhao, Wenying Gao, Chen Huan, Letian Li, Xiang Chen, Hong Wang, Ningyi Jin, Zhao-Qing Luo, Chang Li, Wenyan Zhang

**Affiliations:** 1 Departement of Infectious Diseases, Infectious Diseases and Pathogen Biology Center, Institute of Virology and AIDS Research, Key Laboratory of Organ Regeneration and Transplantation of The Ministry of Education, The First Hospital of Jilin University, Changchun, China; 2 Research Unit of Key Technologies for Prevention and Control of Virus Zoonoses, Chinese Academy of Medical Sciences, Changchun Veterinary Research Institute, Chinese Academy of Agricultural Sciences, Changchun, 130000 Jilin China

**Keywords:** Infection, Drug development, Infectious diseases, Innate immunity

## Abstract

The coronavirus disease 2019 (COVID-19) pandemic caused by severe acute respiratory syndrome coronavirus 2 (SARS-CoV-2) has caused a severe global health crisis; its structural protein envelope (E) is critical for viral entry, budding, production, and induction of pathology which makes it a potential target for therapeutics against COVID-19. Here, we find that the E3 ligase RNF5 interacts with and catalyzes ubiquitination of E on the 63rd lysine, leading to its degradation by the ubiquitin-proteasome system (UPS). Importantly, RNF5-induced degradation of E inhibits SARS-CoV-2 replication and the RNF5 pharmacological activator Analog-1 alleviates disease development in a mouse infection model. We also found that RNF5 is distinctively expressed in different age groups and in patients displaying different disease severity, which may be exploited as a prognostic marker for COVID-19. Furthermore, RNF5 recognized the E protein from various SARS-CoV-2 strains and SARS-CoV, suggesting that targeting RNF5 is a broad-spectrum antiviral strategy. Our findings provide novel insights into the role of UPS in antagonizing SARS-CoV-2 replication, which opens new avenues for therapeutic intervention to combat the COVID-19 pandemic.

## Introduction

The severe acute respiratory syndrome coronavirus 2 (SARS-CoV-2) has caused over 6.65 million deaths globally since the outbreak started in 2019. The genome of this virus encodes 4 structural proteins, spike (S), membrane (M), envelope (E), nucleocapsid (N), and 16 non-structural proteins. The life cycle of SARS-CoV-2 starts with the binding of the S protein to the human angiotensin-converting enzyme 2 (ACE2), which mediates the direct fusion of viral E protein with the host cell membrane and subsequent injection of the viral genome into the host cell. The N protein participates in the packaging of the viral RNA into helical ribonucleoprotein (RNP), which together with the structural proteins move to the endoplasmic reticulum (ER)-Golgi intermediate compartment (ERGIC) where the virions are assembled, matured, and budded into the lumen of secretory vesicular compartments.^1^ Therefore, the structural proteins S, M, E, and N are necessary for SARS-CoV-2 replication. The E protein is highly conserved among different variants and is involved in viral entry, packaging, production, and virulence.^2,3^ The E protein alone has been reported to cause acute respiratory distress syndrome (ARDS)-like pathological damages.^4^ It is also a ligand of TLR2, which drives hyperactive cytokine storms.^5^ There is an urgent need to better understand the molecular mechanism of the interactions between viral proteins and host machinery to facilitate the development of more effective therapies via targeting critical viral proteins.

The RING (really interesting new gene) finger protein 5 (RNF5) is an ER-associated E3 ubiquitin ligase involved in ER-associated degradation (ERAD) for misfolded proteins.^6–9^ In addition to its role in the recognition and ubiquitination of misfolded proteins, RNF5 has been demonstrated to affect the localization of paxillin and other cytoskeletal proteins.^10,11^ RNF5 has also been shown to regulate viral infection by directly targeting antiviral signaling proteins for degradation.^9,12–14^ For example, RNF5 targets the virus-induced signaling adaptor (VISA), the mitochondrial antiviral signaling protein (MAVS), STING, and IRF3 for degradation.^9,14,15^ In addition, some viral proteins have been found to hijack RNF5 to target proteins involved in immunity, leading to the inhibition of innate antiviral responses. Among these, the PB1 protein of influenza A virus (IAV) and the V protein of Newcastle disease virus (NDV) hijack RNF5 to catalyze polyubiquitination of MAVS for its autophagosomal and proteasomal degradation.^12,13^

Here we found that RNF5 recognizes and ubiquitinates the E protein of SARS-CoV-2, leading to its degradation by the ubiquitin-proteasome system (UPS). Furthermore, the RNF5 agonist Analog-1 effectively inhibits SARS-CoV-2 infection in both cells and a mouse infection models. Our results identify RNF5-mediated E protein degradation as a potential therapeutic strategy to treat infections caused by SARS-CoV-2 and related viruses.

## Results

### The E3 ubiquitin ligase RNF5 targets the E protein for proteasomal degradation

**Fig. 1:**
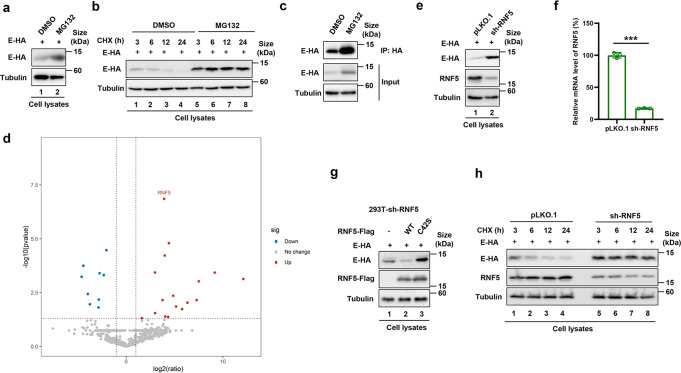
Identification of the E3 ubiquitin ligase RNF5 as a factor involved in degradation of the E protein of SARS-CoV-2. **a** The proteasomal inhibitor MG132 increased the stability of the E protein. **b** MG132 enhanced the half-life of the E protein in cells treated with cycloheximide (CHX). HEK293T cells transfected to express E-HA for 24 h were treated 10 µM MG132 for 10 h. For CHX treatment, cells were harvested at the indicated time points after adding the inhibitor at 50 μg/mL. Cleared lysates resolved by SDS-PAGE were probed by immunoblotting (IB). **c** Immunoprecipitation (IP) of the E protein after MG132 treatment. HEK293T cells transfected to express E-HA were treated with MG132 for 10 h prior to harvest. Lysates were subjected to HA IP and eluents were analyzed by mass spectrometry. **d** Volcano plot of binding proteins with RNF5 in eluents. Black circles represent proteins that were not significantly changed. Red circles represent proteins that potentially interact with the E protein, blue circles represent non-binding proteins. **e** Knockdown of RNF5 increased E stability. **f** mRNA level of silenced RNF5 by RT-qPCR. **g** The E3 ubiquitin ligase activity of RNF5 is required for E degradation. RNF5-silenced HEK293T cells transfected with RNF5-Flag wild-type (WT) or its C42S mutant for 36 h were harvested and analyzed by IB. **h** Knockdown of RNF5 prolonged the half-life of the E protein. HEK293T cells receiving RNF5 silencing or the negative control pLKO.1 were transfected with the E-HA plasmid, and then treated with 50 μg/ml of CHX prior to being harvested at the indicated time points

To investigate whether the structural proteins of SARS-CoV-2 are affected by the UPS, we examined the effect of the proteasome inhibitor MG132 on the protein level of S, E, M, and N and found that treatment by this agent markedly increased the abundancy of E protein but not S, M, or N (Fig. 1a and Supplementary Fig. 1a), suggesting that the E protein is degraded by the UPS. We also determined the role of the UPS in the decay of existing E protein in the absence of de novo protein synthesis. To this end, cells transfected to express the E protein were treated with the protein synthesis inhibitor cycloheximide (CHX) and the kinetics of its decay were examined. Under our experimental conditions, the half-life of the E protein in MG132-treated samples was about 36 h, which was significantly longer than the 6 h observed for the controls (Fig. 1b). The impact of the UPS inhibitor is specific to protein E as it had no effect on the stability of the M protein (Supplementary Fig. 1b).

To identify the E3 ubiquitin ligase responsible for E protein ubiquitination, we expressed HA-tagged E and treated the cells with MG132 prior to co-immunoprecipitation (co-IP) with the HA-specific antibody (Fig. 1c). Proteins co-purified with the HA-tagged E were identified by mass spectrometry. Among the proteins identified, there were some proteasome subunits such as PSMB5, PSMB1, PSMA8 and so on. However, RNF5 was the only E3 ubiquitin ligase and was further investigated (Fig. 1d). Knockdown of RNF5 in HEK293T cells indeed led to stabilization of the E protein (Fig. 1e, f). Furthermore, overexpression of RNF5 in cells receiving the knockdown siRNA destabilized the E protein and such effect did not occur when the catalytically inactive mutant RNF5_C42S_ was expressed (Fig. 1g), while the stability of S, M, or N protein was not affected by RNF5 (Supplementary Fig. 1c). Consistently, in CHX-treated cells, RNF5 knockdown significantly increased the half-life of the E protein at rates comparable to the 6 h observed for non-RNF5 knockdown group (Fig. 1h). Taken together, these results suggest that the E3 ligase RNF5 targets the E protein of SARS-CoV-2 for proteasomal degradation.

### RNF5 interacts with E and catalyzes E protein ubiquitination

RNF5 was originally found to be an ER-anchored ubiquitin ligase involved in ERAD, it was also found to be targeted to the mitochondria.^8,11^ We thus determined which organelle RNF5 and the E protein were targeted by confocal immunofluorescence microscopy analysis. The results showed that RNF5 itself was targeted to both organelles, while the E protein was mostly distributed within the cytoplasm and the nucleus (Supplementary Fig. 2a). Importantly, co-expression of RNF5 and E led to a redistribution of E to the mitochondria but not the ER, which was further verified by its colocalization with the mitochondrial marker COX5 (Supplementary Fig. 2b). Similar results were obtained in cell fractionation experiments. Co-expression with RNF5 markedly increased the amount of E associated with the mitochondria (Supplementary Fig. 2c), suggesting that binding by the E3 ligase RNF5 redistributed E.

**Fig. 2:**
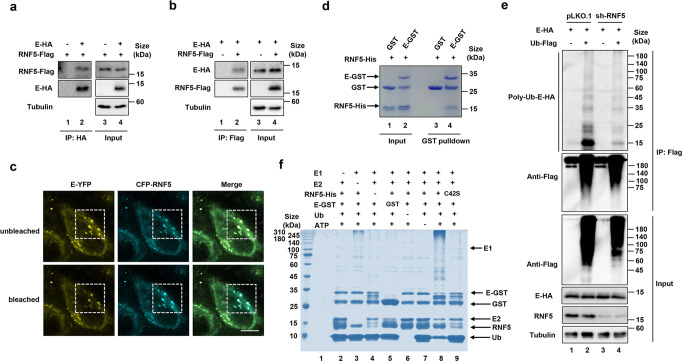
RNF5 interacts with E. HEK293T cells transfected with constructs expressing E-HA and RNF5-Flag were harvested and lysed. Lysates were subjected to HA antibody IP **a** or Flag antibody IP **b**. The precipitates were analyzed by IB with the appropriate antibodies. **c** Interaction between E and RNF5 determined by FRET analysis. Bars, 10 μm. **d** Pulldown of RNF5 by GST-E. Recombinant His6-RNF5 and GST-E were incubated and the potential protein complex was captured with GST Sepharose. **e** Knockdown of RNF5 decreased ubiquitination of E. Lysates of RNF5-silenced HEK293T cells transfected to express E-HA and ubiquitin-Flag were subjected to HA IP and then analyzed by IB. **f** Recombinant RNF5 purified from E. coli catalyzes ubiquitination of E. Dropout reactions with the indicated components were allowed to proceed for 4 h prior to being detected for ubiquitination by Coomassie blue staining

Interactions between an E3 ubiquitin ligase and its substrate are a prerequisite for ubiquitination, we thus examined the binding of RNF5 to the E protein. Binding of these two proteins was readily detectable in reciprocal immunoprecipitation experiments (Fig. 2a, b). Fluorescence resonance energy transfer (FRET) analysis showed that the fluorescence signals of the ECFP-RNF5 fusion became brighter after bleaching the signals of E-YFP (Fig. 2c). Furthermore, incubation of recombinant E-GST with RNF5-His led to pulldown of the latter with GST beads (Fig. 2d), further indicating a direct interaction between these two proteins. We also observed that the E protein was ubiquitinated in HEK293T cells, and knockdown of RNF5 reduced the level of ubiquitination (Fig. 2e). Furthermore, polyubiquitination of E occurred in standard biochemical reactions containing recombinant E1, E2, RNF5, ubiquitin and ATP. No ubiquitination of E protein was detected in reactions receiving the catalytically inactive mutant RNF5_C42S_ (Fig. 2f lanes 8 and 9). The similar results were further conformed by immunoblotting (Supplementary Fig. 2d).

### RNF5 promotes K48-linked ubiquitination of the E protein

**Fig. 3:**
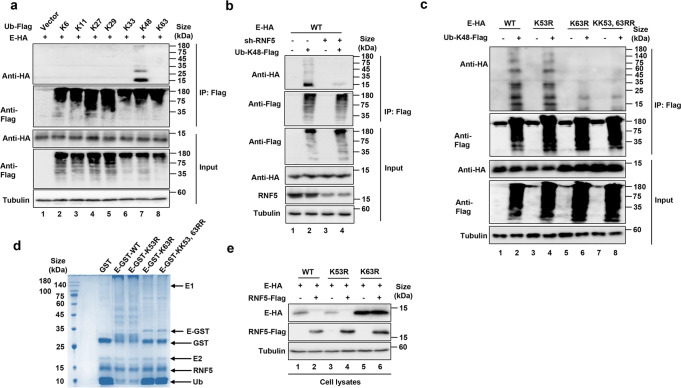
RNF5 catalyzes the formation of K-48 type ubiquitin chains on K63 site of E. **a** E was ubiquitinated via K48-linked but not K6, K11, K27, K29, K33, or K63. **b** knockdown of RNF5 decreased K48-linked ubiquitination of E. Lysates of RNF5-silenced HEK293T cells transfected to express E-HA and ubiquitin-K48-Flag were subjected to HA IP, and then analyzed by IB. **c** RNF5 ubiquitinates E on K63. HEK293T cells transfected to express ubiquitin-K48-Flag and E-HA, E_K53R_, E_K63R_, or E_KK53,63RR_, cell lysates were subjected to Flag IP and the proteins of interest in the precipitates were detected by IB. **d** K63 is the site being ubiquitinated. Standard dropout reactions containing the indicated components were allowed to proceed for 4 h and the proteins were resolved by SDS-PAGE and detected by Coomassie blue staining. **e** The E_K63R_ mutant was recalcitrant to RNF5-mediated degradation. HEK293T cells transfected to co-express RNF5-Flag with E-HA or its K53R or K63R mutants were lysed and the level of relevant proteins was detected by IB

Polyubiquitination can occur in one of the seven lysine residues of ubiquitin, resulting in the formation of different types of ubiquitin chain, which dictate the fate of the modified protein. To investigate the type of polyubiquitin chain formed on the E protein by RNF5, we set up reactions with a series of ubiquitin mutants each containing only one single lysine residue (-K6, -K11, -K27, -K29, -K33, -K48, K63). Ubiquitination of the E protein can only be detected in reactions receiving the K48 ubiquitin mutant (Fig. 3a). Accordingly, knockdown of RNF5 reduced K48-only-linked ubiquitination of the E protein in HEK293T cells (Fig. 3b lanes 2 and 4).

To identify the site of ubiquitination in the E protein induced by RNF5, we constructed arginine substitution mutants at each of its two lysine residues. The degree of ubiquitination of the E_K53R_ mutant was similarly to that of the wild-type protein whereas the E_K63R_ and E_KK53,63RR_ mutants can no longer be modified (Fig. 3c, lanes 4 and 6), suggesting that K63 is the modification site. Consistently, in biochemical reactions, the E_K53R_ mutant was ubiquitinated indistinguishably to that of wild-type protein but ubiquitination of the E_K63R_ and the E_KK53,63RR_ mutants was almost abolished (Fig. 3d), establishing that RNF5 catalyzes ubiquitination on K63 of E. Consistent with its inability to be ubiquitinated by RNF5, the E_K63R_ mutant was resistant to RNF5-mediated degradation (Fig. 3e).

A recent study showed that RNF5-mediated ubiquitination of the M protein of SARS-CoV-2 facilitates its interaction with the E protein and subsequent release of viral particles.^16^ To dissect the relationship among these three proteins, we next examined the effect of M on RNF5-mediated E degradation. We also observed that RNF5 knockdown led to lower levels of M ubiquitination (Supplementary Fig. 3a). Moreover, we found that the presence of M suppressed RNF5-mediated ubiquitination and subsequent degradation of E (Supplementary Fig. 3b, c). Immunoprecipitation with the HA antibody from lysates with similar amounts of HA-M or HA-E co-purified considerably larger amounts of RNF5 in samples expressing HA-M, suggesting that M has a higher affinity for the E3 ligase (Supplementary Fig. 3d), which may explain the observed suppression of E degradation by M. Thus, RNF5 was utilized by both viral protein M and host defense system.

### RNF5 antagonizes SARS-CoV-2 replication

To further investigate the role of RNF5 in SARS-CoV-2 replication, we determined the effects of cellular RNF5 on viral replication. Because endogenous RNF5 in most cell lines effectively degrades the E protein, which makes it difficult to assess the effect of overexpression, we chose Caco2 cells which have a high level of RNF5 for experiments designed to examine the effect of gene knockout, and Vero cells which have low protein abundancy to determine the consequence of RNF5 overexpression (Supplementary Fig. 4a–c).

**Fig. 4:**
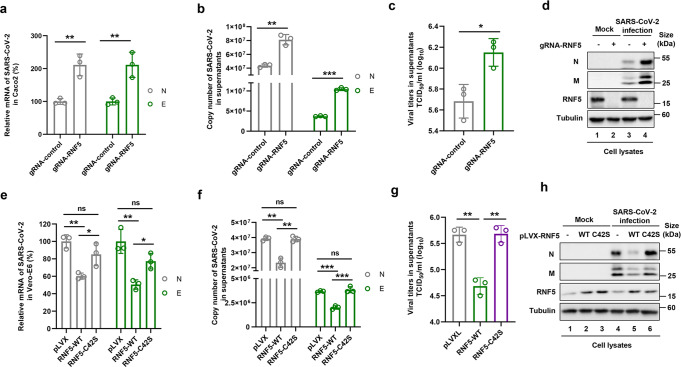
RNF5 inhibits SARS-CoV-2 replication by targeting the E protein for degradation. **a**–**d** RNF5 knockout in Caco2 cells increased viral replication measured by determining mRNA levels of the *N* and *E* genes in cells **a** and in culture supernatants **b**, and viral titers in culture supernatants **c**, and the levels of N and M proteins **d**. Caco2 cells infected with SARS-CoV-2 for 48 h were used to determine viral replication by RT-qPCR or IB analysis. **e**–**h** Overexpression of RNF5 but not the RNF5_C42S_ mutant in Vero-E6 cells reduced viral replication. Vero-E6 cells overexpressing RNF5 WT or the RNF5_C42S_ mutant were infected with SARS-CoV-2 for 48 h, and viral replication in cells was determined by RT-qPCR **e**, and in culture supernatants **f**, viral titers **g** or IB analysis **h**. Statistical significance was analyzed using two-sided unpaired t-tests (NS, no significant, *p < 0.05, **p < 0.01, ***p < 0.001)

We constructed an RNF5-KO cell line from Caco2 cells using the CRISPR/Cas9 technology and examined the role of RNF5 in viral replication (Fig. 4a–c). Wild-type and RNF5-KO cells were infected with SARS-CoV-2 for 48 h, and viral replication was assessed by determining the level of viral mRNA within cells and in culture supernatant and viral titers in culture supernatant. The presence of viral proteins was also detected by immunoblotting. Knocking out RNF5 in Caco2 cells led to an increase in the level of N and M proteins and increased viral titers (Fig. 4a–d). Conversely, overexpression of RNF5 but not the C42S mutant in Vero cells caused a reduction in mRNA of the *N* and *E* genes, decreased viral titers, which was accompanied by a decrease in N and M proteins (Fig. 4e–h). We also employed a biosafety level-2 cell culture system that allowed the production of transcription-and replication-competent SARS-CoV-2 virus-like-particles (trVLPs)^2^ to further confirm the inhibitory effect of RNF5 (Supplementary Fig. 4d). Overexpression of E enhanced the yield of trVLPs. Consistently, reducing cellular level of RNF5 by siRNA phenocopied the effects of E overexpression (Supplementary Fig. 4e, f), further indicating that RNF5-mediated degradation of the E protein inhibits SARS-CoV-2 infection. Taken together, these results indicate that RNF5 inhibits SARS-CoV-2 replication by promoting protein E degradation.

### Higher RNF5 activity suppresses SARS-CoV-2 virulence

A recent study reported that the RNF5 pharmacological activator Analog-1 exerts a potent cytotoxic effect against neuroblastoma and melanoma, probably by promoting the degradation of proteins relevant to the growth of these tumors by activating RNF5.^17^ We thus utilized wild-type cells and cells in which the expression of RNF5 had been silenced by shRNA to test how this agent impacts the stability of E. Analog-1 effectively reduced the stability of E without detectably impacting the protein level of RNF5 (Supplementary Fig. 5a, lanes 1–4). In contrast, similar treatment with Analog-1 of cells in which RNF5 had been silenced did not detectably reduce the protein level of the E protein (Supplementary Fig. 5a, lanes 5–8). Thus, Analog-1 promotes the degradation of E by a mechanism that requires RNF5, likely by inducing its enzymatic activity.

Next we examined the effect of Analog-1 on SARS-CoV-2 replication, wild-type cells and RNF5 knockdown cells treated with different doses of Analog-1 for 24 h, respectively, were infected with SARS-CoV-2. The level of mRNA for the *N* gene was significantly lower in samples receiving the compound than that of untreated samples. Again, the inhibitory effect of Analog-1 on the abundancy of *N* gene mRNA occurs by a mechanism that requires RNF5 (Supplementary Fig. 5b).

**Fig. 5:**
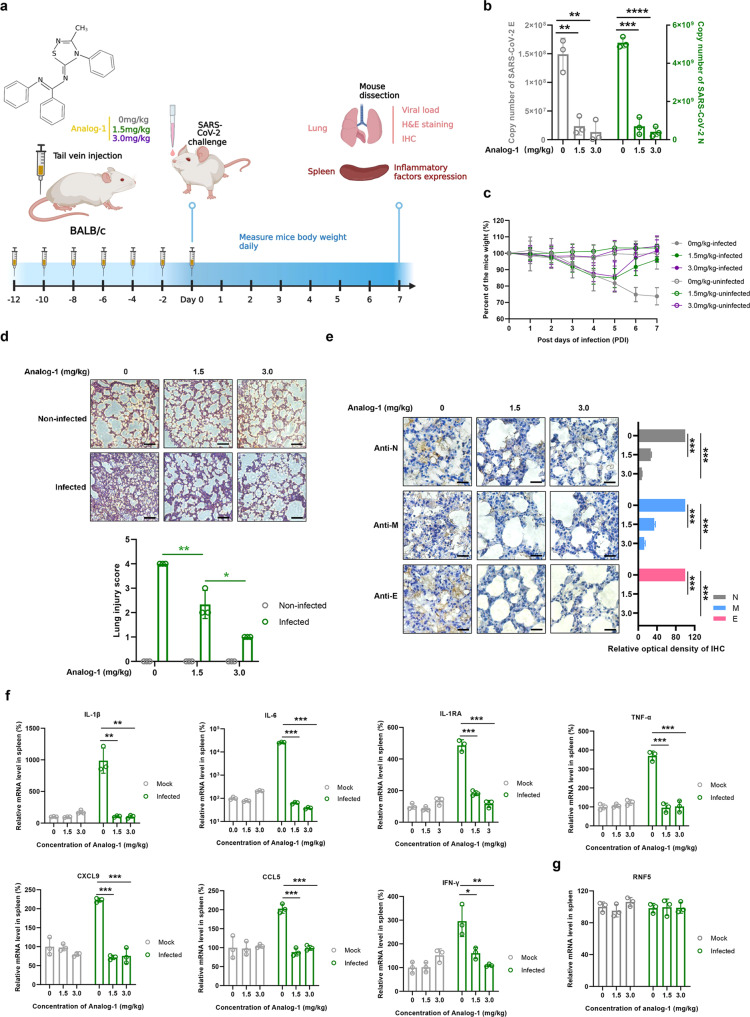
Activation of RNF5 suppresses SARS-CoV-2 virulence in a mouse infection model. **a** BLAB/C mice were treated with Analog-1 at dosage of 0, 1.5 mg/kg or 3.0 mg/kg (20 g mouse, 30 μg or 60 μg) for seven times at 2-day intervals, then infected with the SARS-CoV-2 virus at a dosage of 10^5.5^ TCID_50_/ml via intranasal inoculation. **b** Viral RNA loads of mouse lungs were detected 7 dpi by measuring mRNA levels of *E* and *N* genes. **c** Weight of mice monitored over the experimental durations. **d** Representative images of H&E staining of lungs of differently treated mice. Magnification, ×200. The lung injury score static analysis of HE staining were performed. 0, no injury; 1, <25% injury area; 2, 25–50% injury area; 3, 50–75% injury area; 4, >75% injury area. Bars, 100 μm. **e** The staining of viral N, M and E proteins. Bars, 100 μm. **f** Spleens of each group were evaluated for the expression of cytokines and chemokines by RT-qPCR assay. *N* > 5 for each group. **g** Spleens of each group were evaluated for the expression of RNF5 by RT-qPCR assay. Statistical significance was analyzed using two-sided unpaired t-tests (NS, no significant, *p < 0.05, **p < 0.01, ***p < 0.001)

Finally, we determined the effect of Analog-1 on SARS-CoV-2 virulence in a mouse infection model. BALB/C mice that had been treated with 30 or 60 μg (20 g mouse) of Analog-1 for seven times at 2-day intervals were infected with SARS-CoV-2 virus at a dosage of 10^5.5^ TCID_50_/ml (Fig. 5a). Compared to groups that receiving the solvent as control, the copies of viral *E* and *N* genes in the lung of Analog-1-treated mice were significantly lower. Furthermore, mice receiving the compounds did not exhibit significant weight loss (Fig. 5b, c). Histopathological analysis of the major organs at 7 dpi revealed that Analog-1 treatment alleviated lung lesions such as alveola shrinkage and pulmonary edema typically observed in control mice (Fig. 5d). Consistently, Analog-1 treatment also reduced the abundancy of N, M and E proteins (Fig. 5e).

The E protein of SARS-CoV-2 is known to induce severe inflammation.^4,5^ Indeed, in our disease model, mice infected with SARS-CoV-2 produced high levels of cytokines such as IL-1β, IL-6, IL-1RA, TNF-α, CXCL9, CCL5, and IFN-γ in the spleen. Importantly, Analog-1 treatment significantly reduced the production of these chemokines by similarly infected mice (Fig. 5f). We also observed that SARS-CoV-2 infection had no effect on the expression of RNF5 in the spleen of infected mice (Fig. 5g and Supplementary Fig. 6), which is consistent with the results that RNF5 expression was not affected by SARS-CoV-2 infection in cell culture level (Fig. 4d, h). Together, these results indicate that Analog-1 inhibits SARS-CoV-2 virulence by promoting the degradation of the E protein.

### RNF5 recognizes the E proteins from different variants of SARS-CoV-2

**Fig. 6:**
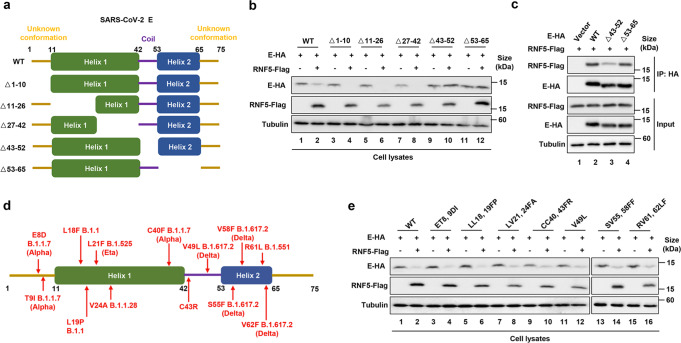
The region of the E protein important for interacting with RNF5. **a** Schematic representation of E deletion mutants. **b** Sensitivity of E mutants to RNF5-mediated degradation. HEK293T cells transfected to express RNF5 and E-HA or its mutants were analyzed for protein levels by IB. **c** The E∆43–52 mutant has considerably lost the ability to bind RNF5. Binding between the E deletion mutants and RNF5 was determined by IP. **d** A schematic of E showing the positions of variations found in SARS-CoV-2 variants. **e** All tested E alleles from different SARS-CoV-2 variants were sensitive to RNF5

To determine the domains in the E protein important for RNF5 recognition, we constructed a series of truncated E protein mutants and examined their sensitivity to RNF5-mediated degradation (Fig. 6a). E mutants lacking amino acids from 43 to 52 or from 53 to 65 were resistant to RNF5-mediated degradation, suggesting that the two regions are important for RNF5 binding (Fig. 6b). Binding assays by IP experiments indicated that only the region that spans 43 to 52 is required for its interactions with RNF5 (Fig. 6c), implying that direct binding to an E3 ligase is not sufficient for ubiquitination, a notion that had been previously noted.^18^

The primary sequences of the E protein are highly conserved in betacoronaviruses, with >90% identity to the protein from isolates of SARS-CoV (Fig. 6d). To examine whether RNF5 also recognizes these E variants, we constructed a series of E alleles that carried each of these variations and examined their sensitivity to RNF5. RNF5 indistinguishably induces the degradation of each of these E protein variants (Fig. 6e). Furthermore, E proteins from SARS-CoV-2 and SARS-CoV share more than 94% identity and both can be degraded by RNF5. In contrast, the E protein of MERS is only 34.15% identical to that from SARS-CoV-2, and this E3 ligase cannot induce its degradation (Supplementary Fig. 7a, b).

### RNF5 expression level is negatively correlated with the severity of the disease caused by SARS-CoV-2

**Fig. 7:**
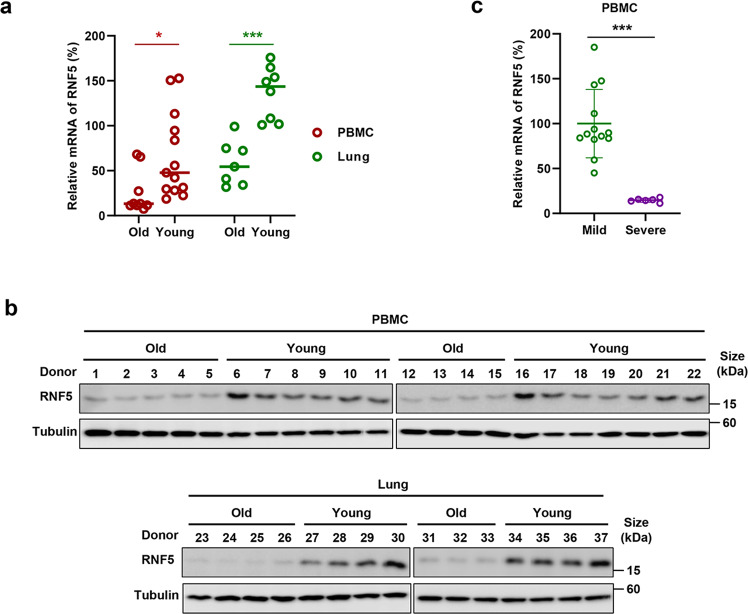
RNF5 expression level is negatively correlated with the severity of the disease caused by SARS-CoV-2. The mRNA level **a** and protein level **b** of RNF5 from samples of PBMC cells and lung tissues of young and older people were determined by RT-qPCR and IB analysis. Results shown are one representative from three independent repeats. **c** The mRNA level of RNF5 from samples of PBMC cells of mild or severe COVID-19 patients was determined by RT-qPCR. Statistical significance was analyzed using two-sided unpaired *t* tests (*p < 0.05, ***p < 0.001)

The clinical manifestations of the disease caused by SARS-CoV-2 infection vary greatly among different age groups. In general, young adults and children often exhibit asymptomatic or mild symptoms, whereas older people with co-morbidities are at higher risk of severe diseases or even death.^19^ We thus examined RNF5 expression in different populations and determine the correlation between RNF5 expression and disease progression. From samples of peripheral blood mononuclear cells (PBMC) and lung tissues, the mRNA level of RNF5 was consistently higher in young people than that found in older populations (Fig. 7a). Similar pattern was observed when the RNF5 protein was examined (Fig. 7b). Importantly, the mRNA level of RNF5 was consistently higher in mild COVID-19 than that found in patients with severe symptoms (Fig. 7c). Although the mechanism underlying the variations in RNF5 expression among the different age groups is unknown, these observations suggest that RNF5 can be used for prognosis of SARS-CoV-2 patients.

## Discussion

The envelope protein is one of the most important structural components of enveloped viruses due to its essential role in viral binding and entry into host cells. The E protein of SARS-CoV-2 is 75-residue transmembrane protein that plays a critical role in its virulence by its ability to induce inflammation, to inhibit the host immune response, and to function as an ion channel.^3–5,16,20,21^ In particular, SARS-CoV-2-E alone causes ARDS-like damages in the lung and spleen in vivo^3^ and is able to induce robust secretion of cytokines and chemokines in macrophages by engaging TLR2.^5^ The structure of E resembles viroporins featuring a pentameric helix bundle surrounding a narrow cationic hydrophilic central pore.^22^ An earlier study demonstrated that recombinant SARS-CoV lacking E showed a significant reduction in viral titer and cannot produce normal progenies.^23^ These essential functions make E an attractive drug target for treating COVID-19. In this study, we identified the E3 ligase RNF5 as a host factor that limits viral propagation by ubiquitination and subsequent degradation of the E protein of SARS-CoV-2. Importantly, we found that the RNF5 activator Analog-1 effectively inhibits SARS-CoV-2 replication in infection models using cultured cells and mice, making it a candidate agent against infections caused by SARS-CoV-2 and related viruses.

The host has evolved effective defense mechanisms to counteract viral propagation. It has long been appreciated that many viruses co-opt the ubiquitination system to target host proteins critical for immunity.^9,14,24–26^ Conversely, ubiquitination and subsequent degradation of viral proteins important for its life cycle is an effective defense mechanism. An emerging class of host inhibitory factors that target the synthesis, trafficking, and/or functions of viral envelope proteins have been identified. One such example is members of the membrane-associated RING-H (MARCH) family of E3 ubiquitin ligases in the RNF family.^25,27–32^ MARCH proteins have broad antiviral activity against diverse enveloped viruses including HIV-1, murine leukemia virus (MLV), VSV-G, Ebola, and SARS-CoV-2.^29,30,33^ In addition, several RNF proteins such as RNF81 (also known as TRIM21) and RNF147 are associated with SARS-CoV-2 infection, but the underlying mechanism of how these enzymes inhibit SARS-CoV-2 replication needs further investigation.^33–35^

Our results here demonstrate that RNF5 inhibits SARS-CoV-2 replication by directly targeting its E protein for degradation. A recent study shows that RNF5 stimulates the interaction between M and E by catalyzing ubiquitination of the K15 residue of M, which facilitates viral release.^16^ The relationship among E, M, and RNF5 appears complex. First, RNF5 induces E ubiquitination and subsequent degradation by the UPS, which appears to be antagonized by M (Supplementary Fig. 3). Second, ubiquitination of M by RNF5 increases its affinity for E. Thus, RNF5-mediated ubiquitination of M and E has opposite effects on SARS-CoV-2 virulence. M seems to promote viral replication by competing for RNF5 activity so that less E will be degraded. Nevertheless, the activity of RNF5 against E is physiologically significant because stimulation of its activity by Analog-1 led to the arrest of viral replication (Fig. 5 and Supplementary Fig. 5).

Consistent with the observation that K63 is the modification site on E, mutations of this residue rendered the protein insensitive to RNF5 (Fig. 3). Interestingly, recent studies reported that K53 and K63 of E can also be acetylated, an event that is involved in its interactions with BRD2 and likely BET proteins.^20,21^ Whether these posttranslational modifications interplay, and if so, how much interplay impacts viral pathogenicity awaits further investigation.

Our observation that both the mRNA and protein levels of RNF5 are higher in young adults than those in older populations, and its mRNA in mild COVID-19 patients is also more abundant than that of patients with severe disease symptoms (Fig. 7), suggests that the abundancy of RNF5 may be used as a biomarker for COVID-19 prognosis. Furthermore, the effectiveness of Analog-1 in lowering the disease burden suggests that this agent or it is further optimized, non-toxic derivatives may be used to treat infections caused by SARS-CoV-2 or related viruses.

## Materials and methods

### Plasmid construction

The cDNA of M protein of SARS-CoV-2 and E proteins of SARS-CoV, SARS-CoV-2, and MERS-CoV linked to an HA tag at N terminals were synthesized and inserted into SalI/BamHI of VR1012^26^ by the Comate Bioscience company (Changchun, CHN). Mutants and truncations of E-HA were constructed by PCR with primers listed in Supplementary Table 1. S-Flag (codon optimized in accordance with the human genome) carrying an Flag tag was inserted into pCDNA6B and N-GFP (N protein was inserted into pEGFP-C1) (BD Biosciences Clontech, catalog no. 6084-1) of SARS-CoV-2 were gifts of Professor Wang Peihui (Shandong University).^36^ Human RNF5 fragments carrying a Flag tag at the N terminal end were amplified from cDNAs of Caco2 cells, and then inserted into SalI/BglII of VR1012. Mutants and truncations of RNF5-Flag were constructed by PCR with primers listed in Supplementary Table 1. For stable expression, wild-type and C42S mutant of RNF5 were individually inserted into pLVX-puro (BD Biosciences Clontech, catalog no. 632164) as *Xho*I/*Bam*HI fragments. The coding region of E of SARS-CoV-2 and human RNF5 were inserted into pCDNA3-YFP (Addgene, catalog no. 13033) and pECFP-C1 (BD Biosciences Clontech, catalog no. 6076-1) respectively for Immunofluorescence (IF) and FRET assays. For protein purification from *Escherichia coIi*, the coding region of E of SARS-CoV-2 with an HA tag at N terminal and human RNF5 were inserted into pGEX-6P-1 (GE Healthcare, catalog no. 27-4597-01) and pET28a (Novagen, catalog no. 69864-3) respectively. Bacterial strains for the production of human E1, E2 (UBC5c), and ubiquitin have been described previously.^37^ Human ubiquitin protein and its mutants carrying an N terminal Flag tag were inserted into VR1012 as SalI/BamHI fragments.

### siRNA, shRNA, and gRNA construction

RNF5-specific siRNA with the following target site was synthesized by RIBO biotechnology company (Guangzhou, CHN). Si-RNF5: 5′-AACGGCAAGAGTGTCCAGTAT-3′. RNF5-specific shRNA with the following target site was cloned in the lentiviral vector pLKO.1-puro (Addgene, catalog no. 8453). shRNF5: 5′- CCGGAACGGCAAGAGTGTCCAGTATCTCGAGATACTGGACACTCTTGCCGTTTTTTTG-3′ and 5′- AATTCAAAAA AACGGCAAGAGTGTCCAGTATCTCGAGATACTGGACACTCTTGCCGTT-3′. CRISPR-control (K010) lentiviral plasmids were obtained from Applied Biological Materials. CRISPR-RNF5 was constructed by PCR with the primers listed in Supplementary Table 1.

### Construction of stably silenced and overexpression cell lines

For cell lines in which the gene of interest was stably silenced, HEK293T cells were co-transfected with shRNF5-pLKO.1 or pLKO.1 plus RRE, REV, and VSV-G expression vectors by using Lipofectamine 2000 (Invitrogen, Carlsbad, CA, USA). Packed lentiviral particle collected 48 h after transfection was used to infect HEK293T cells for 48 h and then puromycin (3 μg/ml, Sigma, St. Louis, MO, USA) was used to select stable cell lines. For stable overexpressing cell lines, pLVX-RNF5 or pLVX were introduced and the cell lines were similarly selected and screened.

### Construction of gene knockout cell lines

Lentiviral particles carrying the gRNA and Cas9 produced in HEK293T cells were used to infect Caco2 cells. Puromycin was added at 10 μg/ml 48 h after transduction and the selection was allowed to proceed for 72 h. Surviving cells diluted in tissue culture medium were used to obtain single cell-derived clones with the desirable gene knockout. RNF5-defective clones were identified by immunoblotting and one was chosen for subsequent experiments.

### Cell culture and viruses

HEK293T (American Type Culture Collection [ATCC], Manassas, VA, USA, catalog no. CRL-11268), Caco2 (ATCC catalog no. HTB-37), A549 (ATCC, catalog no. CCL-185), Vero-E6 (ATCC, catalog no. CRL-1586) and Hela (ATCC, catalog no. CRM-CCL-2) cells were cultured as monolayers in Dulbecco’s modified Eagle’s medium (DMEM) (Hyclone, Logan, UT, USA), which was supplemented with 10% heat-inactivated (56 °C, 30 min) fetal calf serum (FCS, GIBCO BRL, Grand Island, NY, USA), and maintained at 37 °C with 5% CO_2_ in a humidified atmosphere. SARS-CoV-2 isolate CHN/Beijing_IME-BJ01/2020 (GenBank access no. MT291831.1) and mouse-adapted SARS-CoV-2/C57MA14 variant (GenBank: OL913104.1), originated from human throat swabs, was propagated in Vero-E6 cells in DMEM supplemented with 2% FBS, and titered using the median tissue culture infectious dose (TCID_50_) assay.^38^ All experiments of infectious SARS-CoV-2 were conducted under Biosafety Level 3 facilities.

### Transfection and infection

DNA transfections were carried out by Lipofectamine 3000 Reagent (Invitrogen, catalog no. L3000-008). siRNA transfections were carried out by Lipofectamine RNAiMAX Reagent (Invitrogen, catalog no. 13778150) according to the manufacturer’s instructions.

For SARS-CoV-2 infection, cells grown to 70% confluence in the six-well plates were washed twice with phosphate-buffered saline (PBS) and incubated with SARS-CoV-2 isolate at 37 °C for 1 h at an MOI of 0.1. Plates were gently agitated at 15-min intervals to facilitate adsorption. After adsorption, the virus-containing medium was replaced with fresh medium containing 2% FCS, followed by incubation at 37 °C in 5% CO_2_ for the indicated durations.

### Antibodies and immunoblotting

Tissues from donors, transfected and infected HEK293T, Caco2, or Vero-E6 cells were harvested and boiled in 1× loading buffer (0.08 M Tris, pH 6.8, with 2.0% SDS, 10% glycerol, 0.1 M dithiothreitol and 0.2% bromophenol blue) followed by separation on a 13.5% polyacrylamide gel. Proteins in the samples were transferred onto a polyvinylidene fluoride (PVDF) membrane for IB analysis. The membranes treated with sealer solution for 30 min were incubated with indicated primary antibodies, and followed by a corresponding horse radish peroxidase (HRP)-conjugated secondary antibody (Jackson Immunoresearch, West Grove, USA, catalog no. 115-035-062 for anti-mouse and 111-035-045 for anti-rabbit) diluted 1:20000 respectively. At last, the proteins on the PVDF membranes were visualized by the ultra-sensitive ECL chemiluminescence detection kit (Proteintech, Rosemont, IL, USA, catalog no. B500024).

The following antibodies were used in this study: RNF5 polyclonal antibody (pAb) (Sangon Biotec, Shanghai, CHN, catalog no. D225282), SARS-CoV-2 nucleocapsid antibody (GeneTex, Irvine, CA, USA, catalog no. GTX635679), SARS-CoV-2 Membrane antibody (GeneTex, catalog no. GTX636246), SARS-CoV-2 Envelop antibody (GeneTex, catalog no. GTX136046), anti-myc pAb, (Proteintech, catalog no. 16286-1-AP), anti-hemagglutinin (anti-HA) pAb (Invitrogen, Carlsbad, USA, catalog no. 71-5500), anti-tubulin mAb (Abcam, Cambridge, Cambridgeshire, UK, catalog no. ab11323), anti-Flag mAb (Sigma, Saint Louis, USA, catalog no. F1804), anti-GFP mAb (Abcam, catalog no. ab1218). For confocal microscopy, anti-COX5A pAb (Sangon Biotec, catalog no. D261450), SelectFX® Alexa Fluor® 488 Endoplasmic Reticulum Labeling Kit (Invitrogen, catalog no. S34200), Goat anti-Rabbit IgG (H + L) Highly Cross-Adsorbed Secondary Antibody, Alexa Fluor™ Plus 555 (Invitrogen, catalog no. A32732) and goat anti-Rabbit IgG (H + L) Highly Cross Adsorbed Secondary Antibody, Alexa Fluor Plus 568 (Invitrogen, catalog no. A-11011) were used.

ER Isolation Kit (Sigma, catalog no. ER0100) was used for ER isolation. Mitochondria Isolation Kit (Beyotime Institute of Biotechnology, Shanghai, CHN, catalog no. C3601) was used for mitochondria isolation.

### RNA extraction and RT-qPCR

For RT-qPCR, intracellular and viral RNA was extracted from various organizations or cell lines used in this study with Trizol reagent (Invitrogen), RNase inhibitor (New England BioLabs, Ipswich, MA, USA), and diethyl pyrocarbonate (DEPC)-treated water. The cDNAs were generated by a High-Capacity cDNA Reverse Transcription kit (Applied Biosystems, Carlsbad, CA, USA) and random or oligo (dT) 18 primers according to the supplier’s instructions. Reverse transcription was carried out in a 20 μl volume, which contained 1 μg RNA extracted from the above samples. RT-qPCR was carried out by the RealMaster Mix (SYBR Green Kit, Takara, Shiga, Japan) and primers designed targeting sequences of hRNF5, SARS-CoV-N, SARS-CoV-E and hGAPDH on an Mx3005P instrument (Agilent Technologies, Stratagene, La Jolla, CA, USA). The RT-qPCR assay was carried out in a 20 μl volume consisting of 1 μl of 5 μmol/L of each oligonucleotide primer, 2 μg of cDNA templates, and 9 μl of 2.5× RealMaster Mix/×20 SYBR Green solution, which contained the HotMaster Taq DNA Polymerase. Amplifications of the target fragment were carried out as the following steps: initial activation of the HotMaster Taq DNA Polymerase at 95 °C for 2 min, and then followed with 40 cycles of 95 °C for 15 s, 57 °C for 15 s and 68 °C for 20 s. All oligonucleotide primers used for qPCR were listed in Supplementary Table 1.

### Co-Immunoprecipitation (co-IP) assay

For IP of proteins with an HA tag, HEK293T cells transfected with indicated plasmids for 48 h, were harvested and washed twice with the cold PBS, followed by ultrasonication in a lysis buffer [PBS containing 1% Triton X-100 and complete protease inhibitor cocktail (Roche, Basel, Basel-City, SUI, catalog no. 11697498001) and 10 μM MG132 (Abcam, catalog no. ab141003)]. Cell lysates cleared by centrifugation at 10,000 × *g* for 30 min at 4 °C were mixed with anti-HA agarose beads (Roche, catalog no. 11867423001) and incubated at 4 °C for 4 h on an end-over-end rocker. The beads which bound proteins were then washed six times with the cold wash buffer (20 mM Tris-HCl, pH 7.5, 100 mM NaCl, 0.1 mM EDTA, 0.05% Tween-20). Proteins drop-down by beads were analyzed by IB. For Ub-Flag and RNF5-Flag IP, cell lysates were mixed with protein-G agarose beads (Roche, catalog no. 11243233001) and anti-Flag monoclonal antibody.

### Mass spectrometry

HEK293T were transfected with SARS-CoV-2-E-HA for 36 h, then treated with MG132 or DMSO for 12 h prior to harvest. Co-IP assay were performed with HA beads (Roche, catalog no. 11867423001), and the elution was analyzed by mass spectrum. Mass spectrum analysis were performed by the national center for protein science (Beijing, CHN).

### Pulldown assays

SARS-CoV-2-E with a GST tag and RNF5 with a His tag were transformed into the *E. coli* Transetta (DE3) strain (Transgen Biotech, Beijing, CHN) respectively. After induction with 0.5 M IPTG at 16 °C overnight, cells suspended with the A buffer (20 mM Tris-HCl and 50 mM NaCl) were lysed by ultrasonication. And then lysates were cleared by centrifugation at 12,000 × *g* for 30 min at 4 °C. On the other hand, the Ni^2+^ sepharose was incubated with 0.1 M NiSO_4_ for 30 min at room temperature and washed with the A buffer. After above operations, the supernatants of the lysates were harvested, and purified with Ni^2+^ sepharose or GST sepharose (General Electric Company, Boston, MA, USA) respectively at 4 °C. SARS-CoV-2-E-GST and RNF-his, which have been concentrated and replaced with the A buffer with Amicon Ultra-15 (Millipore, Billerica, MA, USA, catalog no. UFC901024), were mixed as indicated and incubated with GST Sepharose for 4 h at 4 °C. After six times wash with PBS, the sepharoses were treated with 1× loading buffer and boiled. And then the targeting proteins were detected by Coomassie blue staining.

### FRET analysis

Hela cells seeded in six-well glass-bottom plates were transfected with E-YFP (1 μg) and ECFP-RNF5 (1 μg), then were treated with 10 μM MG132 to avoid the degradation for 12 h prior to fixing, following fixing in 4% paraformaldehyde at room temperature for 15 min and washing with PBS for three times. Fluorescent images of samples were then acquired with Olympus FV 3000 confocal imaging system.

### Confocal microscopy

For localization of E or RNF5 alone, or colocalization of E with RNF5, Hela cells were transfected with E-YFP or RNF5-CFP alone, or V E-YFP and RNF5-CFP for 48 h. The cells were fixed in 4% paraformaldehyde at room temperature for 15 min, washed with PBS, permeabilized in 0.1% Triton X-100 for 5 min, washed in PBS, blocked in 2% BSA for 1 h, and then incubated at room temperature for 2 h with mouse anti-PDI antibody, which was contained in SelectFX® Alexa Fluor® 488 Endoplasmic Reticulum Labeling Kit, at 1:1000 or rabbit anti-COX5A antibody at 1:200. Following a wash, cells were incubated at room temperature with corresponding Highly Cross Adsorbed Secondary Antibody at 1:1000 for 1 h. After being washed with cold PBS, cells were analyzed by using a laser scanning confocal microscope (FV 3000, Olympus, Tokyo, JPN).

### In vitro ubiquitination assays

For in vitro ubiquitination, E1-his, UBC5c-his, Ub-K48-his, RNF5-his, RNF5-C42S-his, and E-GST were purified by Ni^2+^ sepharose or GST sepharose, respectively, then concentrated and replaced with buffer A by Amicon Ultra-15 at 4 °C. After added with 10% (v/v) glycerol, purified proteins were subpackage and stored in a refrigerator at −80 °C. Protein concentration was determined using the Bradford assay with BSA as the standard. And then, 100 nM E1, 100 nM UBC5c, 1 μM RNF5, 2.5 μM Ub-K48, and 1 μM E-GST were added into 50 μl reaction system as indicated which contained 50 mM Tris-HCl (pH 7.5), 1 mM DTT. 0.5 mM ATP and 5 mM MgCl_2_. After 2 h reaction performed at 37 °C, target proteins were detected with Coomassie blue staining.

### Mouse lines and infection

BALB/C mice were purchased from Charles River Laboratories (Beijing, CHN). All welfare and experimental procedures were carried out strictly in accordance with the Guide for the Care and Use of Laboratory Animals and the related ethical regulations. All efforts were made to minimize animal suffering. The mice were randomly divided into six groups, and each group contained eight mice. Three groups were treated with the dosage of 0, 1.5, 3.0 mg/kg Analog-1 for seven times every two days via tail vein injection, then infected with a mouse-adapted SARS-CoV-2/C57MA14 variant (GenBank: OL913104.1)^39^ at a dosage of 10^5.5^ TCID_50_/ml via intranasal challenge. No Analog-1 treatment and SARS-CoV-2 infection were used as a negative control. 1.5 and 3.0 mg/kg of Analog-1 treatment only was used to evaluate the toxicity of Analog-1.

### Immunohistochemical analysis (IHC)

A total of 18 mice (three mice in each group) were anesthetized, lung and spleen were harvested. Lungs were fixed with 3.7 % paraformaldehyde solution for 2 days. Then, all of the lungs were dehydrated via an ethanol gradient, clarified through dimethylbenzene, and embedded in paraffin, and 4-μm sections were obtained for hematoxylin and eosin (H&E) staining. Histopathological analysis of the lungs was performed under a light microscope. The endogenous peroxidase activity of the tissues was inhibited by treatment with hydrogen peroxide (2.5 %). Amount of SARS-CoV-2 N, M, and E proteins in the lung were detected by SARS-CoV-2 nucleocapsid monoclonal antibody (mAb) (GeneTex, catalog no. GTX635679), SARS-CoV-2 Membrane antibody (GeneTex, catalog no. GTX636246), SARS-CoV-2 Envelope antibody (GeneTex, catalog no. GTX136046), and a Streptavidin-Peroxidase Anti-Rabbit IgG kit (Maixin, Fuzhou, CHN, catalog no. KIT-9706).

### Statistical analysis

The detailed statistical analysis has been described in figure legends. All data are expressed as the mean ± standard deviations (SDs). Statistical comparisons between the two groups were made using a Student’s *t* test. Significant differences are indicated in the figures as follows: *P < 0.05, **P < 0.01, and ***P < 0.001. *P* values of <0.05 are considered to represent a statistically significant difference. ns stands for no significance.

### Ethics statement

The collection of blood samples and tissues from hospitalized patients was approved by the ethics committee of the First Hospital of Jilin University (21K105-001) and complied with the guidelines and principles of WMA Declaration of Helsinki and the Department of Health and Human Services Belmont Report. Informed consent was signed by all research participants. PBMCs were from 22 Healthy Volunteer Blood Donors (9 old donors, 52–78 years old, and 13 young donors, 26–35 years old) and 13 mild and 6 severe COVID-19 patients (Supplementary Table 2). Pericarcinomatous tissues from 15 donors with lung cancer (7 old donors, 53–76 years old, and 8 young donors, 24–35 years old). All animal experiments were approved by the ethics committee of Research Unit of Key Technologies for Prevention and Control of Virus Zoonoses, Chinese Academy of Medical Sciences, Changchun Veterinary Research Institute, Chineses Academy of Agricultural Sciences (IACUC of AMMS-11-2020-006).

## Supplementary information

Supplementary figures and tables

## Data Availability

The authors declare that [the/all other] data supporting the findings of this study are available within the paper [and its supplementary information files]. The mass spectrometry proteomics data have been deposited to the ProteomeXchange Consortium (http://proteomecentral.proteomexchange.org) via the iProX partner repository with the dataset identifier PXD039140.
