# Correction To: The E3 ligase RNF5 restricts SARS-CoV-2 replication by targeting its envelope protein for degradation

**DOI:** 10.1038/s41392-023-01374-y

**Published:** 2023-02-27

**Authors:** Zhaolong Li, Pengfei Hao, Zhilei Zhao, Wenying Gao, Chen Huan, Letian Li, Xiang Chen, Hong Wang, Ningyi Jin, Zhao-Qing Luo, Chang Li, Wenyan Zhang

**Affiliations:** 1 Departement of Infectious Diseases, Infectious Diseases and Pathogen Biology Center, Institute of Virology and AIDS Research, Key Laboratory of Organ Regeneration and Transplantation of The Ministry of Education, The First Hospital of Jilin University, Changchun, China; 2 Research Unit of Key Technologies for Prevention and Control of Virus Zoonoses, Chinese Academy of Medical Sciences, Changchun Veterinary Research Institute, Chinese Academy of Agricultural Sciences, Changchun, 130000 Jilin China

**Keywords:** Infection, Drug development, Infectious diseases, Innate immunity

Correction to: *Signal Transduction and Targeted Therapy* 10.1038/s41392-023-01335-5, published online 03 February 2023

In the process of collating the raw data, the authors noticed 7 inadvertent mistakes occurred in Figs. 3a, 3b, 3e, 5d, 6b, 6d and 6e that need to be corrected after online publication of the article.^1^ The correct data are provided as follows. The key findings of the article are not affected by these corrections. The original article has been corrected.

1. The plus signs in Fig. 3a should line up with the lane.
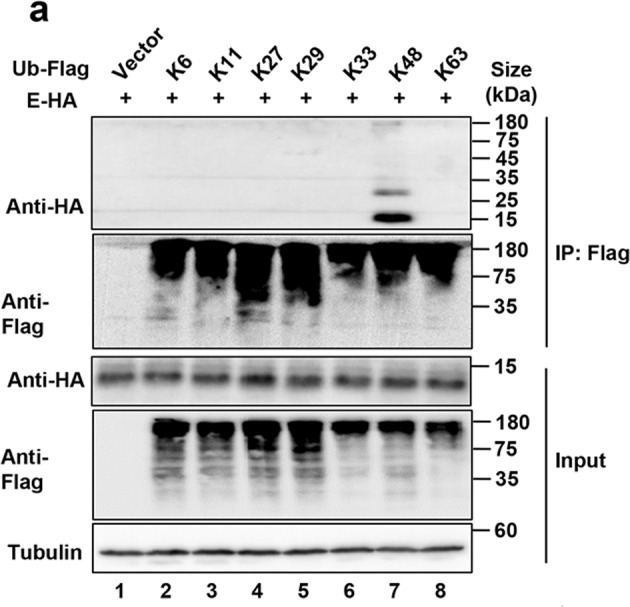
  Fig. 3a E was ubiquitinated via K48-linked but not K6, K11, K27, K29, K33 or K63.
2. The plus signs and minus signs in Fig. 3b should line up with the lane.
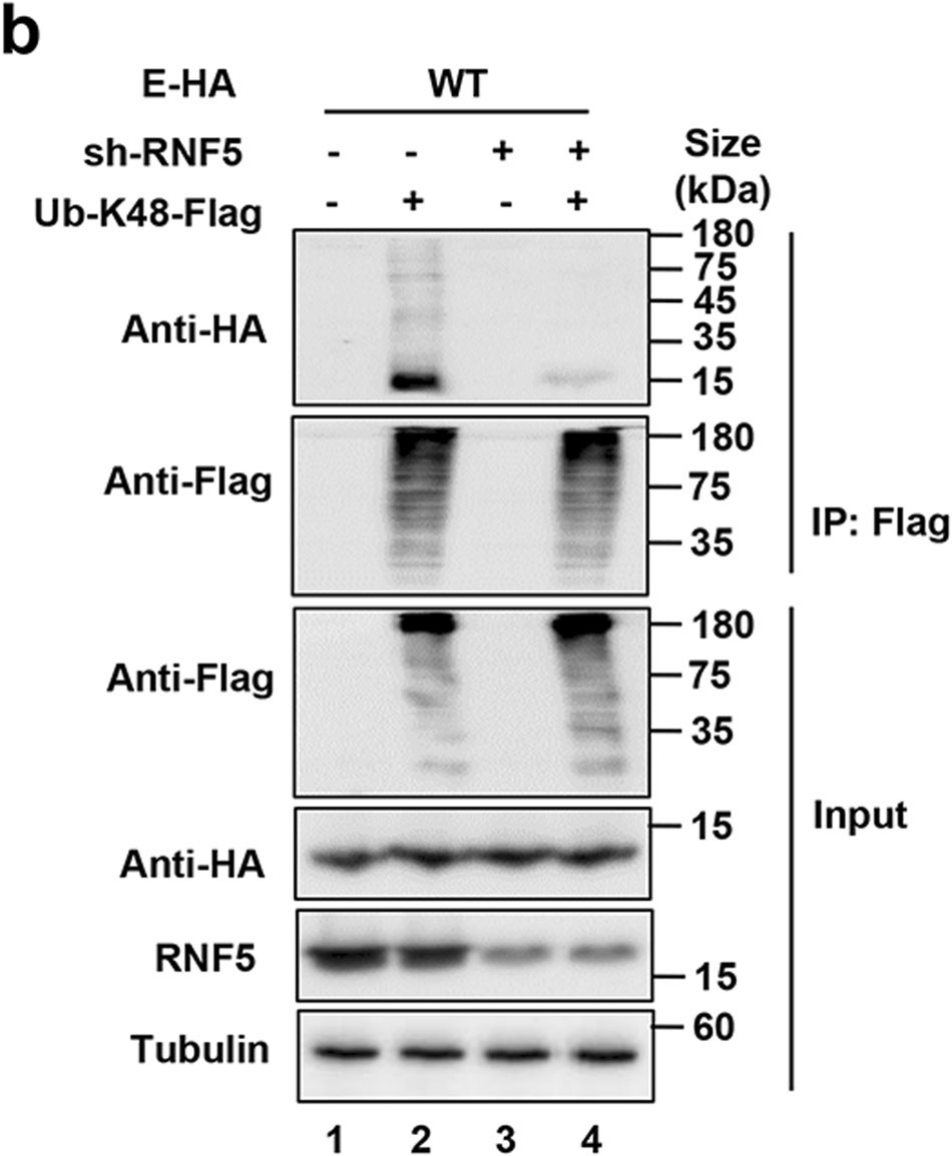
  Fig. 3b Knockdown of RNF5 decreased K48-linked ubiquitination of E.
3. The plus signs and minus signs in Fig. 3e should line up with the lane.
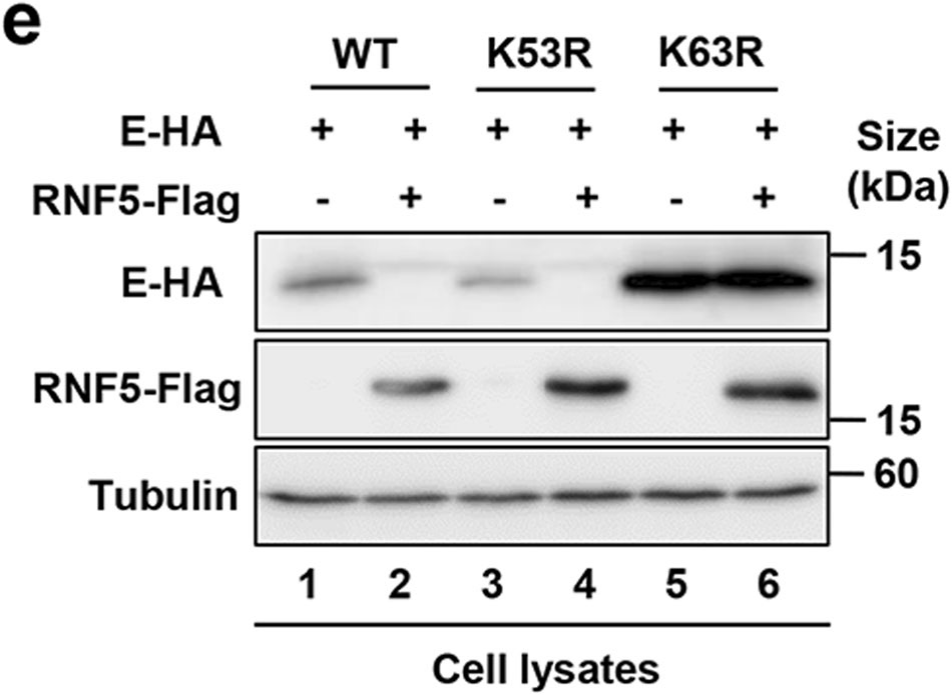
  Fig. 3e The EK63R mutant was recalcitrant to RNF5-mediated degradation.
4. Due to our negligence, the picture of another field of vision of Analog-1-1.5mg/kg-non-infected was mistakenly used as the picture of Analog-1-0mg/kg-non-infected in Fig. 5d. The correct picture of Analog-1-0mg/kg-non-infected is shown below.
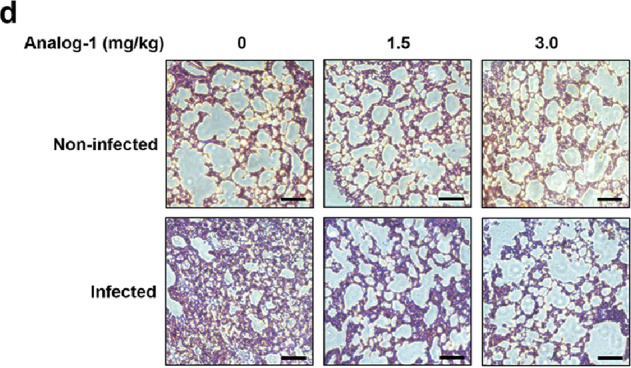
  Fig. 5d Representative images of H&E staining of lungs of differently treated mice.
5. The plus signs and minus signs in Fig. 6b should line up with the lane.
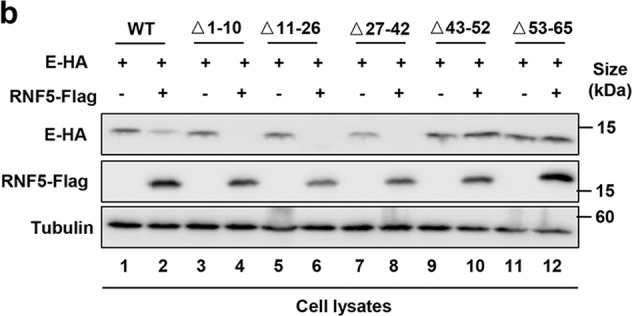
  Fig. 6b Sensitivity of E mutants to RNF5-mediated degradation.
6. The first mutation site found in Alpha strain marked as “E8D B.1.1.7 (Alpha)” was overlapped with the arrows in Fig. 6d.
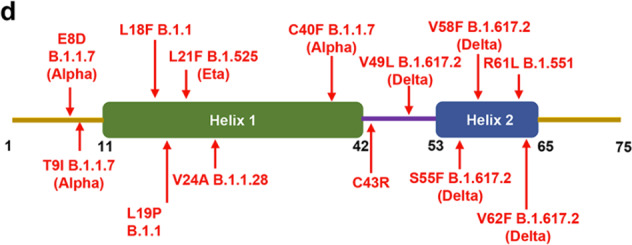
  Fig. 6d A schematic of E showing the positions of variations found in SARS-CoV-2 variants.
7. The plus signs and minus signs in Fig. 6e should line up with the lane.

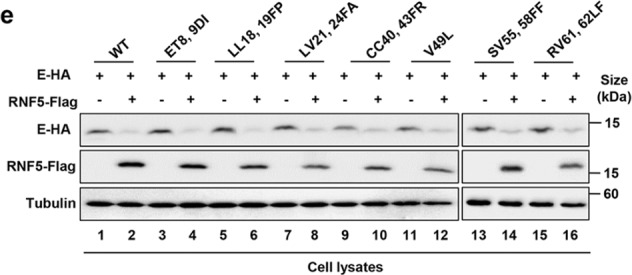


Fig. 6e All tested E alleles from different SARS-CoV-2 variants were sensitive to RNF5.

## References

[1] Li Z (2023). The E3 ligase RNF5 restricts SARS-CoV-2 replication by targeting its envelope protein for degradation. Signal Transduct. Target. Ther..

