# Supplementary material for: The E3 ligase RNF5 restricts SARS-CoV-2 replication by targeting its envelope protein for degradation
Source: Signal Transduct Target Ther. 2023 Feb 3;8:53. doi: 10.1038/s41392-023-01335-5 (PMC9897159; doi:10.1038/s41392-023-01335-5)
Supplement: Supplementary file 1 — Supplementary figures and tables [file 41392_2023_1335_MOESM1_ESM.docx]

Supplementary Materials for

**The E3 ligase RNF5 restricts SARS-CoV-2 replication by targeting its envelope protein for degradation**

Zhaolong Li^1^, Pengfei Hao^2^, Zhilei Zhao^1^, Wenying Gao^1^, Chen Huan1, Letian Li^2^, Xiang Chen^1^, Hong Wang^1^, Ningyi Jin^2^, Zhao-Qing Luo^1^*, Chang Li^2^* and Wenyan Zhang^1^*

1Departement of Infectious Diseases, Infectious Diseases and Pathogen Biology Center, Institute of Virology and AIDS Research, Key Laboratory of Organ Regeneration and Transplantation of The Ministry of Education, The First Hospital of Jilin University; 2Research Unit of Key Technologies for Prevention and Control of Virus Zoonoses, Chinese Academy of Medical Sciences, Changchun Veterinary Research Institute, Chineses Academy of Agricultural Sciences, Changchun 130000, Jilin, China

*Correspondence:

Wenyan Zhang ([zhangwenyan@jlu.edu.cn](mailto:zhangwenyan@jlu.edu.cn)), Chang Li ([lichang78@163.com](mailto:lichang78@163.com)), and Zhao-Qing Luo (luoz@jlu.edu.cn)

**This PDF file includes:**

Figures. S1 to S7

Tables S1 to S2

Figure. S1.

**
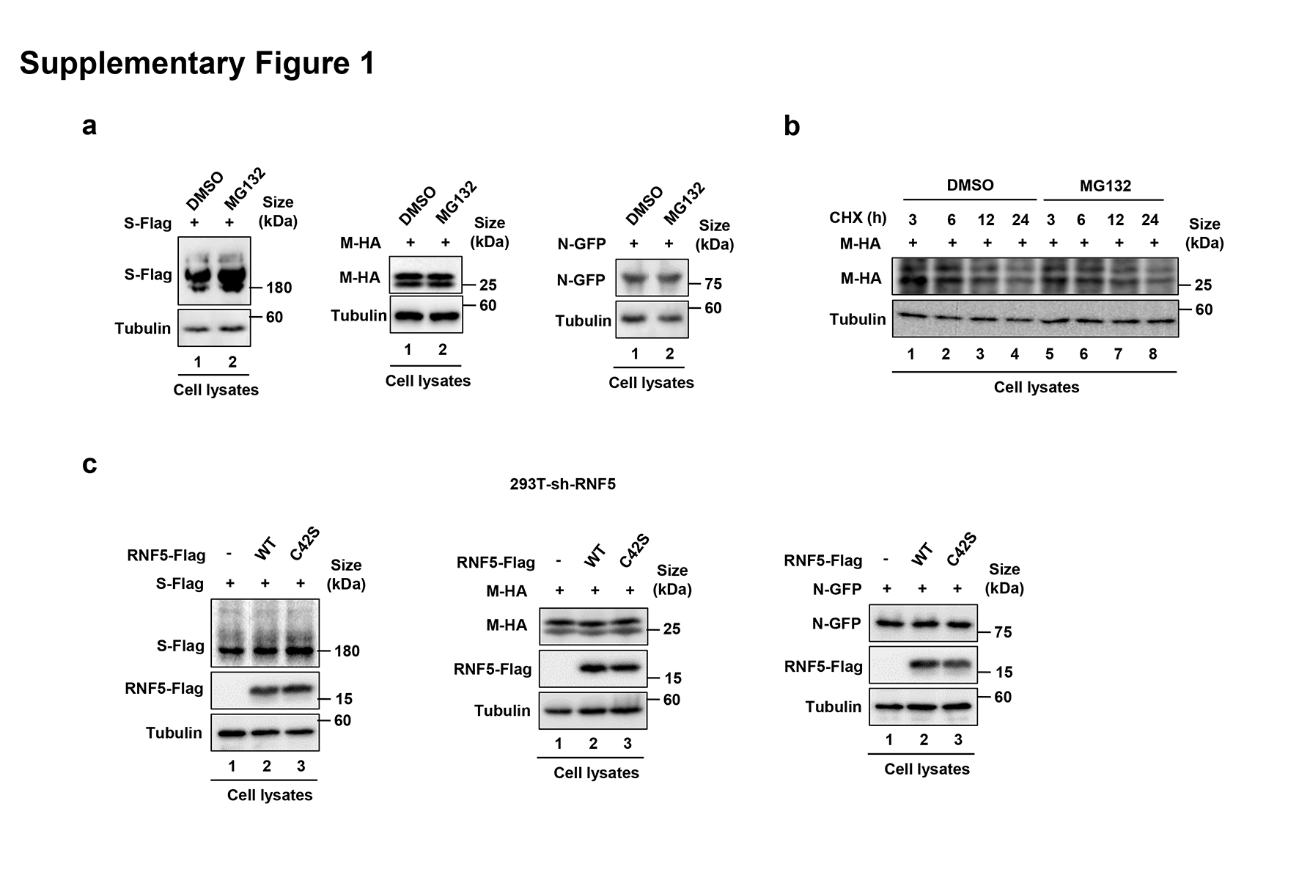
**

**Fig. S1 SARS-CoV-2 structural proteins S, M and N were not degraded by proteasomal pathway.** (a) The proteasomal inhibitor MG132 did not stabilize the S, M or N protein. (b) MG132 had no effect on the half-life of the S, M or N protein in cells treated with cycloheximide (CHX). (c) Overexpression of RNF5 or its C42S mutant in RNF5-silencing cells did not alter the protein levels of S, M or N.

Figure. S2.


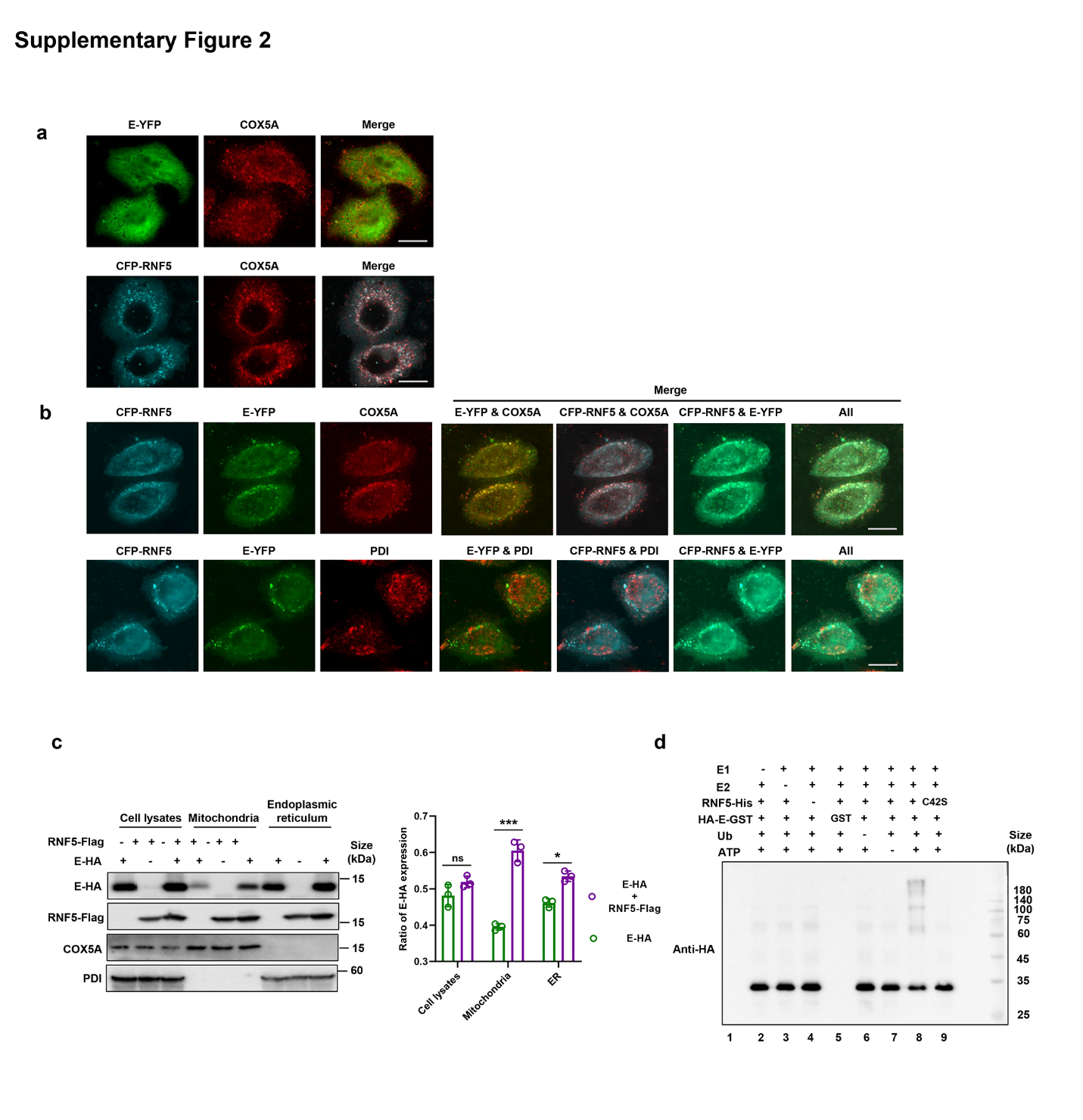


Figure. S2. E causes cellular redistribution of RNF5. (a) Confocal immunofluorescence microscopy analysis of RNF5 or E in Hela cells. COX5 is used as a marker for the mitochondria and PDI is an ER marker. Bars, 10μm. (b) Colocalization of RNF5 and E in the mitochondria. Bars, 10μm. (c) Co-expression of RNF5 caused more E redistribution in mitochondria. (d) HA-E-GST in Fig. 2F were detected by immunoblotting with anti-HA antibody.

Figure. S3.


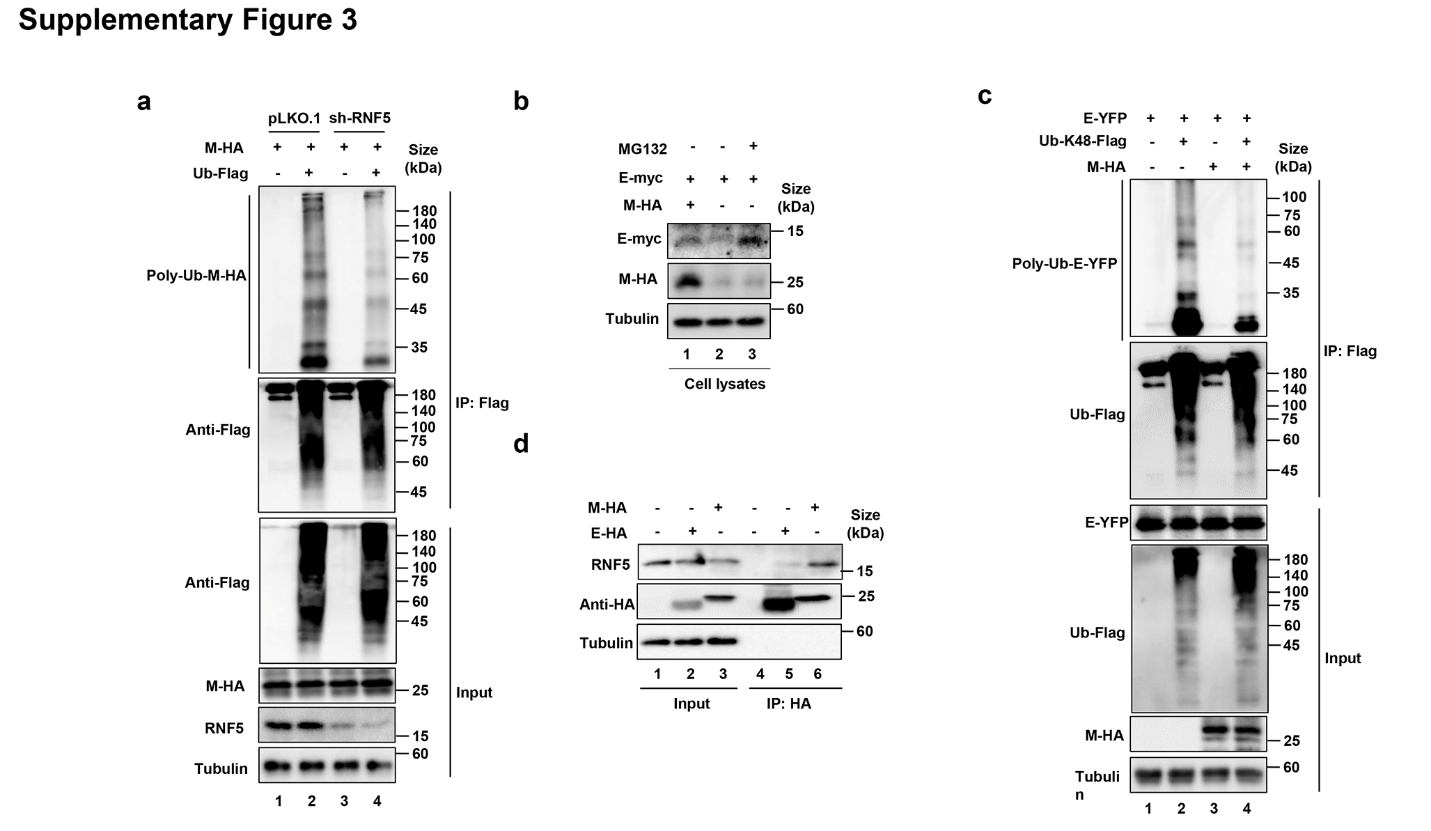


Figure. S3. The M protein of SARS-CoV-2 interferes with RNF5-mediated E degradation. (a) Ubiquitination of M by RNF5. Lysates of RNF5-silenced HEK293T cells transfected to express M-HA and ubiquitin-Flag were subjected to HA IP and the precipitates were used to detect the protein level of M. (b M suppressed the degradation of E protein by RNF5. (c) M reduced RNF5-mediated ubiquitination of E. Lysates of HEK293T cells transfected to express M-HA together with E-YFP and ubiquitin-K48-Flag were subjected to Flag IP, the products were analyzed by IB. (d) The binding of RNF5 to E is weaker than M. Lysates of HEK293T cells transfected to express E-HA or M-HA were subjected to HA IP and the co-purified RNF5 was detected by IB.

Figure. S4.


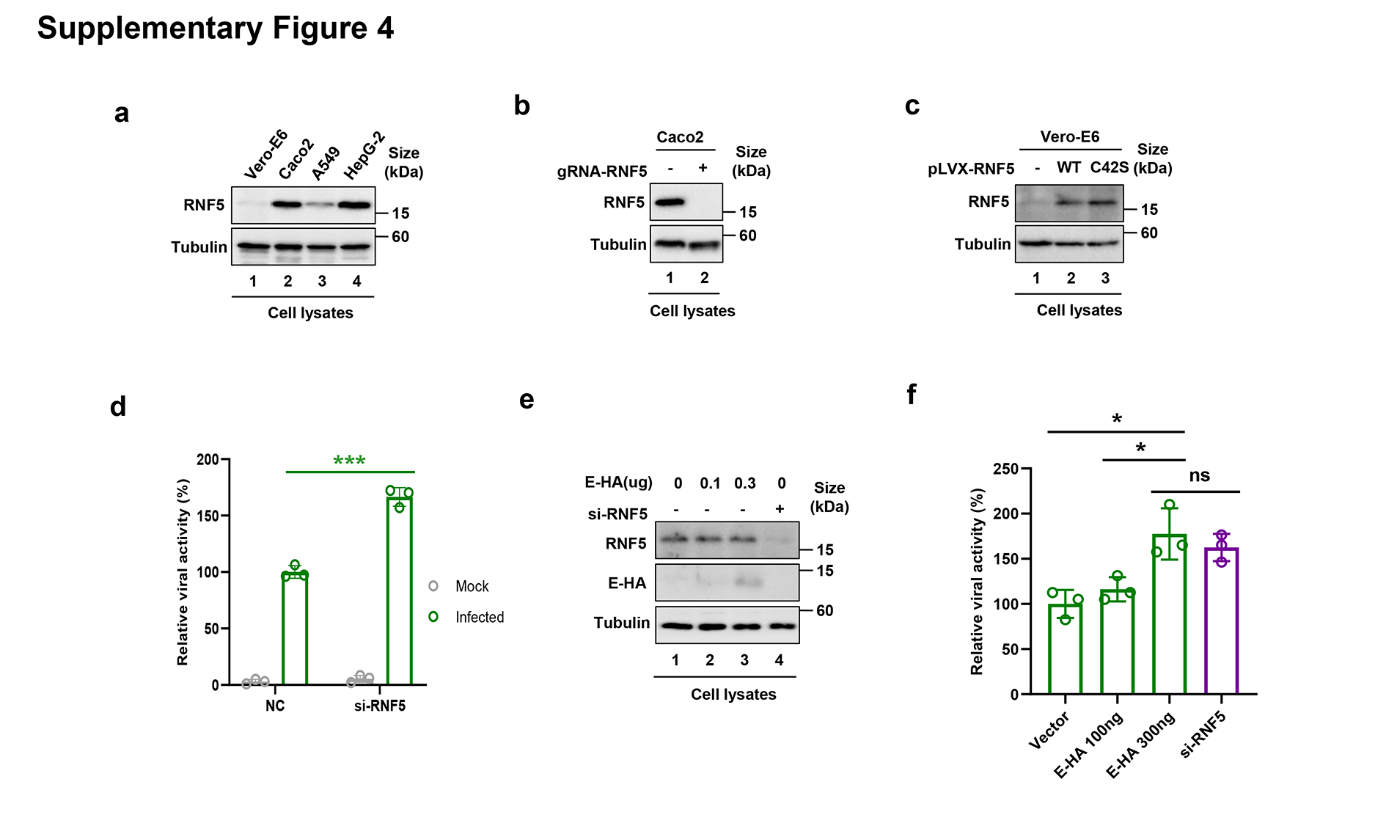


**Figure. S4. RNF5 antagonizes SARS-CoV-2 infection measured by using the trVLP system.** (a) The protein level of RNF5 in various cell lines. (b) Construction of a RNF5 knockout Caco2 cell line. (c) Construction of a Vero-E6 cell line that overexpresses RNF5. (d) Silencing RNF5 led to increased SARS-CoV-2 replication. Caco2-Nint cells were transfected with RNF5 siRNA for 24 h, then were infected with SARS-CoV-2 trVLPs at an MOI of 0.1 for 48 h. The infection was analyzed using flow cytometry to detect eGFP positive cells.

Figure. S5.


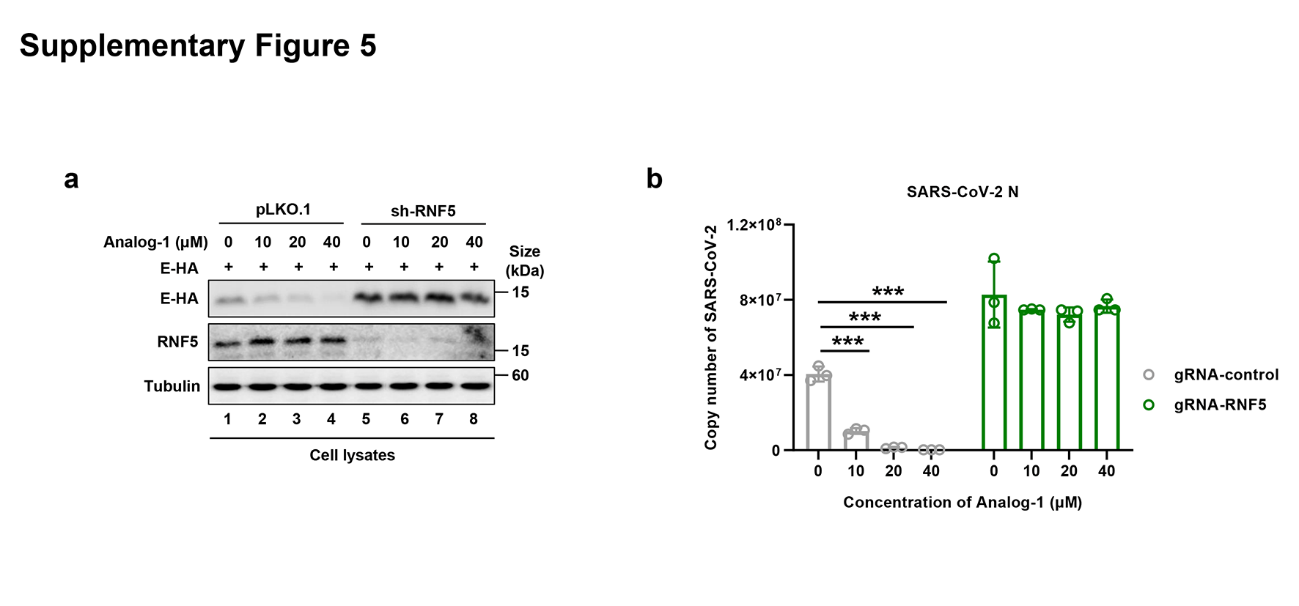


**Figure. S5.** **Activation of RNF5 suppresses SARS-CoV-2 replication in cells.** (a) Analog-1 destabilized the E protein by a mechanism that requires RNF5. HEK293T cells receiving RNF5 silencing or the control were transfected to express E-HA for 24 h, samples treated with different dosages of Analog-1 for another 24 h were harvested for IB analysis for the proteins of interest. (b) Analog-1-induced suppression of SARS-CoV-2 replication required RNF5. Wild-type cells and RNF5 knockout cells treated with different dosages of Analog-1 for 24 h, respectively were infected with SARS-CoV-2. The mRNA level of viral N gene was determined by RT-qPCR 48 h post-infection.

Figure. S6.


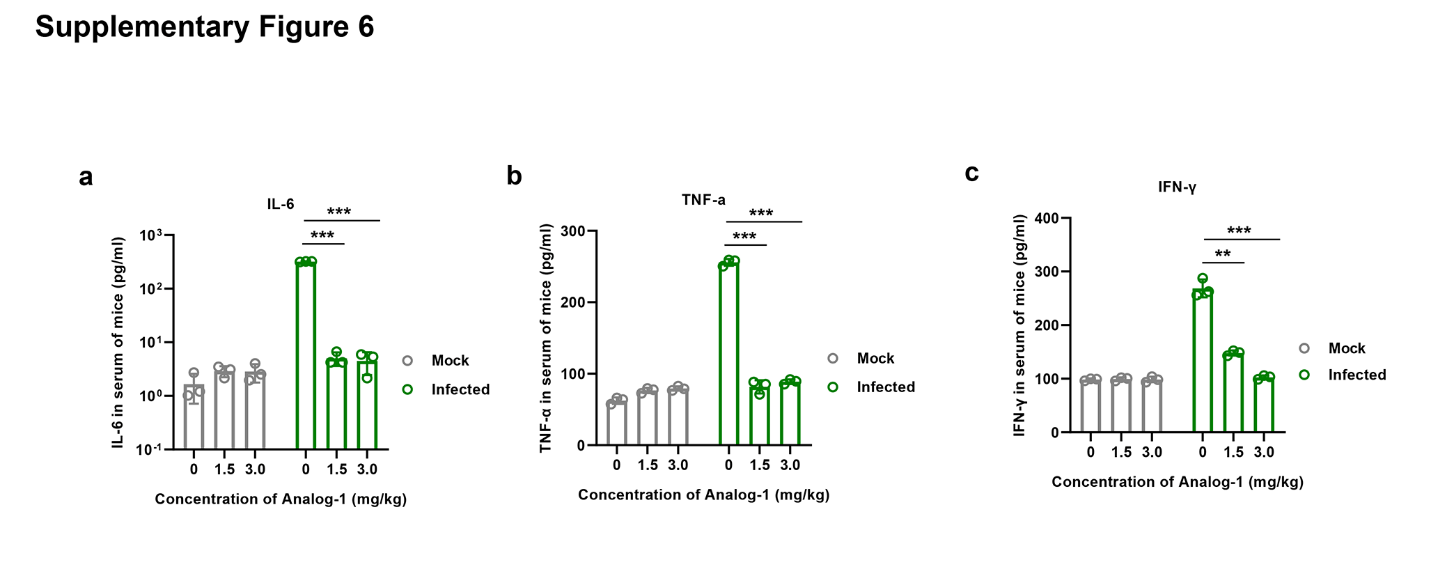


**Figure. S6. Analog-1 reduce the expression of cytokines and chemokines.** IL-6, TNF-α, and IFN-γ (a-c) by ELISA. Statistical significance was analyzed using two-sided unpaired t-tests (NS, no significant, **, p < 0.01, ***, p<0.001).

Figure. S7.


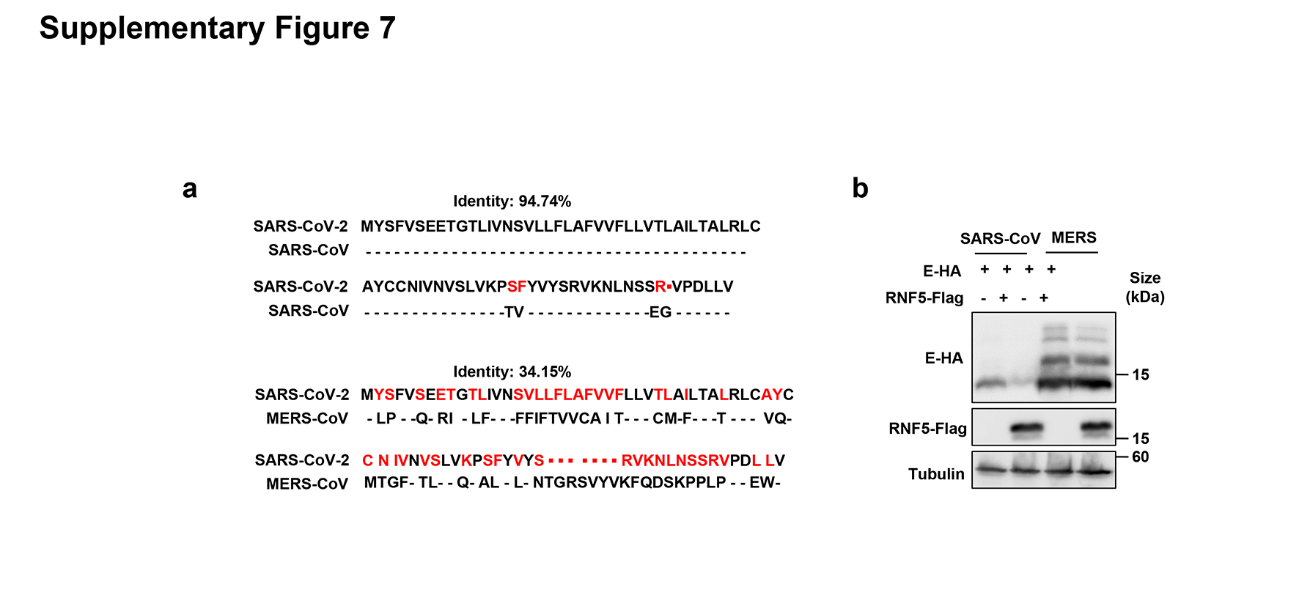


**Figure. S7. The E protein of SARS-CoV but not MERS is sensitive to RNF5-mediated degradation.** (a) The alignment of SARS-CoV-2, SARS-CoV, and MERS. (b) The E protein of SARS-CoV but not MERS can be degraded by RNF5.

Table S1 Primers and siRNA used in this study

| Primer | Sequence (5’-3’) | Enzyme site | Purpose |
| --- | --- | --- | --- |
| E-K53R-F | CGTGAGTCTTGTAAGACCTTCTTTTTACG | None | E-K53R mutant |
| E-K53R-R | CTTACAAGACTCACGTTAACAATATTG | None | E-K53R mutant |
| E-K63R-F | GTTTACTCTCGTGTTAGAAATCTGAATTCTTC | None | E-K63R mutant |
| E-K63R-R | CTAACACGAGAGTAAACGTAAAAAG | None | E-K63R mutant |
| E-△1-10-F | TCCCAGATTACGCGACGTTAATAGTTAATAGC | None | E-△1-10 truncation |
| E-△1-10-R | GTCGCGTAATCTGGGACGTCGTAAG | None | E-△1-10 truncation |
| E-△11-26-F | CGGAAGAGACAGGTTTGCTAGTTACACTAG | None | E-△11-26 truncation |
| E-△11-26-R | AACCTGTCTCTTCCGAAACGAATG | None | E-△11-26 truncation |
| E-△27-42-F | GCTTTCGTGGTATTCTGCTGCAATATTGTTAAC | None | E-△27-42 truncation |
| E-△27-42-R | CAGAATACCACGAAAGCAAGAAAAAG | None | E-△27-42 truncation |
| E-△43-52-F | CGATTGTGTGCGTACAAACCTTCTTTTTACG | None | E-△43-52 truncation |
| E-△43-52-R | TGTACGCACACAATCGAAGCGCAGTAAG | None | E-△43-52 truncation |
| E-△53-65-F | TAACGTGAGTCTTGTAAATTCTTCTAGAGTTC | None | E-△53-65 truncation |
| E-△53-65-R | ATTTACAAGACTCACGTTAACAATATTG | None | E-△53-65 truncation |
| SalI-flag-RNF5-F | GCGTCGACATGGACTACAAGGACGACGATGACAAGGGCAGCAGCGGAGGA | SalI | RNF5-flag-VR1012 |
| RNF5-BglII-R | GAAGATCTTCAAATACTGAGCAGCCAAAAAAAG | BglII | RNF5-flag-VR1012 |
| RNF5-C42S-F | CTGTGGTCAGTGTGAGTGGCCACCTGTAC | None | RNF5-C42S mutant |
| RNF5-C42S-R | TCACACTGACCACAGCTTCCCGAG | None | RNF5-C42S mutant |
| sh-RNF5-F | CCGGAACGGCAAGAGTGTCCAGTATCTCGAGATACTGGACACTCTTGCCGTTTTTTTG | AgeI & EcoRI | sh-RNF5-pLKO.1 |
| sh-RNF5-R | AATTCAAAAAAACGGCAAGAGTGTCCAGTATCTCGAGATACTGGACACTCTTGCCGTT | AgeI & EcoRI | sh-RNF5-pLKO.1 |
| gRNA-RNF5-F | AAAGGACGAAACACCGCTCGCGATTTGGCCCTTCGGTTTTAGAGCTAGAAATAG | None | gRNA-RNF5 |
| gRNA-RNF5-R | CGGTGTTTCGTCCTTTCCACAAGAT | None | gRNA-RNF5 |
| si-RNF5 | AACGGCAAGAGUGUCCAGUAU | None | Shutting down RNF5 |
| N-SARS-CoV-2-RT-F | GGGGAACTTCTCCTGCTAGAAT | None | Real-time qPCR |
| N-SARS-CoV-2-RT-R | CAGACATTTTGCTCTCAAGCTG | None | Real-time qPCR |
| Probe for N-SARS-CoV-2 | FAM-TTGCTGCTGCTTGACAGATT-TAMRA | None | Real-time qPCR |
| E-SARS-CoV-2-RT-F | CGATCTCTTGTAGATCTGTTCTC | None | Real-time qPCR |
| E-SARS-CoV-2-RT-R | ATATTGCATTGCAGCAGTACGCACA | None | Real-time qPCR |
| Probe for E-SARS-CoV-2 | FAM-ACACTAGCCATCCTTACTGCGCTTCG-BHQ1 | None | Real-time qPCR |
| Homo-RNF5-RT-F | TGGCCATGTCTTCATCAGTG | None | Real-time qPCR |
| Homo-RNF5-RT-R | GTTTTTAATCTGGGATCCTGG | None | Real-time qPCR |
| Homo-GAPDH-RT-F | CCCATCACCATCTTCCAGG | None | Real-time qPCR |
| Homo-GAPDH-RT-R | TTCTCCATGGTGGTGAAGAC | None | Real-time qPCR |
| Mus-IL-1β-RT-F | TGTGGAGAAGCTGTGGCAG | None | Real-time qPCR |
| Mus-IL-1β-RT-R | CAGCAGGTTATCATCATCATC | None | Real-time qPCR |
| Mus-IL-6-RT-F | CACTTCACAAGTCGGAGGC | None | Real-time qPCR |
| Mus-IL-6-RT-R | TTTGTATCTCTGGAAGTTTCAG | None | Real-time qPCR |
| Mus-IL-1RA-RT-F | ACCTGAGAAACAACCAGCTC | None | Real-time qPCR |
| Mus-IL-1RA-RT-R | CTTGGCACAAGACAGGCAC | None | Real-time qPCR |
| Mus-TNF-α-RT-F | CTCAAAGACAACCAACTAGTG | None | Real-time qPCR |
| Mus-TNF-α-RT-R | TGGTATGAGATAGCAAATCGG | None | Real-time qPCR |
| Mus-CXCL9-RT-F | ATCAGCACCAGCCGAGGC | None | Real-time qPCR |
| Mus-CXCL9-RT-R | CCGGATCTAGGCAGGTTTG | None | Real-time qPCR |
| Mus-CCL5-RT-F | ACCATATGGCTCGGACACC | None | Real-time qPCR |
| Mus-CCL5-RT-F | ACACTTGGCGGTTCCTTCG | None | Real-time qPCR |
| Mus-IFN-γ-RT-F | AGTCTCTTCTTGGATATCTGG | None | Real-time qPCR |
| Mus-IFN-γ-RT-R | ATGACGCTTATGTTGTTGCTG | None | Real-time qPCR |
| Mus-GAPDH-RT-F | CAGTGGCAAAGTGGAGATTG | None | Real-time qPCR |
| Mus-GAPDH-RT-R | TCCCGTTGATGACAAGCTTC | None | Real-time qPCR |

Table S2 Patients clinical information in this study

| Patient number | Age | Gender | Severity |
| --- | --- | --- | --- |
| 1 | 62 | Female | Severe |
| 2 | 68 | Male | Severe |
| 3 | 77 | Female | Severe |
| 4 | 75 | Female | Severe |
| 5 | 50 | Male | Severe |
| 6 | 75 | Male | Severe |
|  | Average: 67.83 | Male (50.00%) |  |
| 7 | 65 | Male | Mild |
| 8 | 47 | Female | Mild |
| 9 | 54 | Female | Mild |
| 10 | 71 | Female | Mild |
| 11 | 49 | Female | Mild |
| 12 | 47 | Male | Mild |
| 13 | 66 | Male | Mild |
| 14 | 62 | Male | Mild |
| 15 | 51 | Male | Mild |
| 16 | 26 | Male | Mild |
| 17 | 46 | Female | Mild |
| 18 | 52 | Male | Mild |
| 19 | 65 | Female | Mild |
|  | Average: 53.92 | Male (53.84%) |  |
